# Association Analysis and Identification of ZmHKT1;5 Variation With Salt-Stress Tolerance

**DOI:** 10.3389/fpls.2018.01485

**Published:** 2018-10-12

**Authors:** Zhilei Jiang, Guangshu Song, Xiaohui Shan, Zhengyi Wei, Yanzhi Liu, Chao Jiang, Yu Jiang, Fengxue Jin, Yidan Li

**Affiliations:** ^1^Jilin Provincial Key Laboratory of Agricultural Biotechnology, Agro-Biotechnology Research Institute, Jilin Academy of Agricultural Sciences, Changchun, China; ^2^College of Plant Sciences, Jilin University, Changchun, China; ^3^School of Life Sciences, Jilin Agricultural University, Changchun, China

**Keywords:** high-affinity potassium transporter (*HKT*), natural variation, stress-associated gene, salt tolerance, maize (*Zea mays*)

## Abstract

The high-affinity potassium transporter (HKT) genes are essential for plant salt stress tolerance. However, there were limited studies on HKTs in maize (*Zea mays*), and it is basically unknown whether natural sequence variations in these genes are associated with the phenotypic variability of salt tolerance. Here, the characterization of *ZmHKT1;5* was reported. Under salt stress, *ZmHKT1;5* expression increased strongly in salt-tolerant inbred lines, which accompanied a better-balanced Na^+^/K^+^ ratio and preferable plant growth. The association between sequence variations in *ZmHKT1;5* and salt tolerance was evaluated in a diverse population comprising 54 maize varieties from different maize production regions of China. Two SNPs (A134G and A511G) in the coding region of *ZmHKT1;5* were significantly associated with different salt tolerance levels in maize varieties. In addition, the favorable allele of *ZmHKT1; 5* identified in salt tolerant maize varieties effectively endowed plant salt tolerance. Transgenic tobacco plants of overexpressing the favorable allele displayed enhanced tolerance to salt stress better than overexpressing the wild type *ZmHKT1;5*. Our research showed that *ZmHKT1;5* expression could effectively enhance salt tolerance by maintaining an optimal Na^+^/K^+^ balance and increasing the antioxidant activity that keeps reactive oxygen species (ROS) at a low accumulation level. Especially, the two SNPs in *ZmHKT1;5* might be related with new amino acid residues to confer salt tolerance in maize.

**Key Message:** Two SNPs of *ZmHKT1;5* related with salt tolerance were identified by association analysis. Overexpressing *ZmHKT1;5* in tobaccos showed that the SNPs might enhance its ability to regulating Na^+^/K^+^ homeostasis.

## Introduction

Soil salinity is a serious abiotic stress restricting crop productivity severely, as more than 800 million hectares of land have been salt-affected in the world. Furthermore, the amount is still rising. Salinity has become a growing threat to sustainability of agricultural (Zhu, 2001; Munns, 2005; Munns and Tester, 2008). One effective approach to this problem is to breed salt-tolerant crop cultivars to improve the productivity of salt-stressed soils (Yamaguchi and Blumwald, 2005).

Two types of stress are often imposed by high salinity to plants: ionic toxicity and osmotic shock (Zhu, 2001; Munns, 2005; Munns and Tester, 2008). Plants have developed a series of mechanisms to respond salt stress, such us regulating Na^+^/K^+^ homeostasis and Na^+^ exclusion (Ren et al., 2005; Munns and Tester, 2008). Many genes associated with enhanced salt-tolerance have been identified in different plant species. The high-affinity potassium transporter (HKT) genes, belonging to the Trk/Ktr (K^+^ transporter)/HKT transporter family, are known to be responsible for regulating transportation of Na^+^ and K^+^ in higher plants (Munns and Tester, 2008; Horie et al., 2009; Corratge-Faillie et al., 2010). The *TaHKT2;1* gene from wheat (*Triticum aestivum*) was the first HKT gene found in plants (Schachtman and Schroeder, 1994). Recent research has indicated that members of HKT family should be involved in the exclusion of Na^+^ from leaves in crops (Very and Sentenac, 2003; Rodriguez-Navarro and Rubio, 2006; Huang et al., 2008; Haro et al., 2010). In rice (*Oryza sativa*), *SKC1*, a quantitative trait locus controlling rice salt tolerance was identified to encode *OsHKT1;5* (Ren et al., 2005). Similarly, HKT is also essential for salt tolerance in durum wheat (*Triticum durum*). *Nax1* and *Nax2*, which were identified as *TmHKT1;4-A2* and *TmHKT1;5-A*, restrict the accumulation of Na^+^ in wheat leaves and have the potential to enhance salt tolerance (James et al., 2011). In barley (*Hordeum vulgare* L.), association analysis disclosed that Na^+^ and K^+^ transport was mainly controlled by *HvHKT1* and *HvHKT2* (Qiu et al., 2011). In *Sorghum bicolor*, *SbHKT1;4* expression maintained a better balanced Na^+^/K^+^ ratio in salt-tolerant accessions under salt stress (Wang et al., 2014). Consequently, HKT family is involved in Na^+^/K^+^ homeostasis and is expected to show broad importance in salinity tolerance.

Although HKT is very important, it is basically unknown whether natural variation in HKT genes is associated with levels of salt tolerance in maize and whether some favorable alleles of HKT genes can be identified and utilized to breed salt-tolerant crop varieties.

Maize not only is an important crop in the world, but also is selected for some association studies (Wisser et al., 2011; Yan et al., 2011; Riedelsheimer et al., 2012). Candidate gene association analysis has been successfully applied to discover allelic variety in genes controlling aluminum tolerance, drought tolerance, b-carotene content, kernel size, and fatty acid content in maize with proper statistical models (Krill et al., 2010; Li et al., 2010a,b, 2011; Yan et al., 2010; Liu et al., 2013;Mao et al., 2015; Wang et al., 2016; Xiang et al., 2016). More salt-resistant maize varieties are being screened out, although maize is generally regarded as a salt-sensitive crop (Cramer, 1992; Geilfus et al., 2010; Shahzad et al., 2015; Zorb et al., 2015). In consequence, inspecting the association between the genetic variation in maize HKT genes and salt tolerant performance in maize varieties will help to not only promote the genetic improvement of salt tolerance but also to broaden our understanding of HKT gene family.

In this study, *ZmHKT1;5* was cloned and analyzed to determine its phylogenetic relationships to other *HKTs* and its expression pattern under the salt stress. Importantly, the association between the genetic variation in *ZmHKT1;5* and salt tolerance was evaluated using a diverse population comprising 54 maize varieties from different maize production regions of China. A significant association between *ZmHKT1;5* variation and salt-tolerance at the seedling stage was discovered. Moreover, a favorable allele of *ZmHKT1;5* in response to salt stress was tested, and transgenic tobacco plants were generated to verify its function. The results showed that this favorable allele of *ZmHKT1;5* mediated physiological processes that were involved in salt stress tolerance.

## Materials and Methods

### Phylogenetic Tree Construction

Full-length amino acid sequences of 40 HKTs identified in 28 kinds of plant (**Supplementary Table S1**), including maize, rice, wheat, barley, sorghum and Arabidopsis, were aligned using the ClustalX 1.83 with default pairwise and multiple alignment parameters. The phylogenetic tree was constructed based on this alignment using the neighbour joining (NJ) method in MEGA version 5^1^ with the following parameters: Poisson correction, pairwise deletion, uniform rates, and bootstrap (1000 replicates). The ZmHKT proteins were named according to their placement in the phylogenetic tree.

### Plant Growth and Salt Treatment

Fifty-four inbred lines originating from different eco-geographic regions were obtained from the Jilin Academy of Agricultural Sciences (**Supplementary Table S2**). Maize seeds were surface-sterilized with 70% alcohol for 1 min and then 3% NaClO for 10 min, thoroughly rinsed with distilled water and germinated on filter paper wetted with distilled water in plates at 28°C for 3 days. Twelve germinated seeds of each genotype were transplanted to enriched soil (turf and vermiculite in a ratio of 1:1) pots. The seedlings were grown in a growth chamber. The temperature was maintained at 22°C during the day and 20°C at night, while the daily photoperiod was set at 14 h. Light intensity was set at 70 μmol/m^2^s. A salinity treatment was applied to the soil-grown plants at the 3-leaf seedling stage by watering with 200 mL 100 mM NaCl every day. The time point for salt tolerance evaluation was determined by the characterization of salt tolerance among all the genotypes, e.g., the wilting rate. Typically, leaves of most inbred lines should be wilted after about 7 days salt treatment. Watering was then resumed in order to recover the surviving plants. After rehydration for 3 days, the survival rate of each genotype was assessed. The phenotypic data for salt treatments were obtained from no less than three independent replicated experiments.

### *ZmHKT1;5* Gene Sequence and Association Analysis With Salt Tolerance among 54 Maize Inbred Lines

The full-length of *ZmHKT1;5* gene, including the 5′ and 3′ untranslated regions (UTR) sequences, was amplified in different maize inbred lines. The primers were designed using Primer3 web version 4.0.0 (FW: 5′-ACGCTCCCCACAGAAACTAA, REV: 5′-GAGTGAGCGACGAGAACCTA) by using the B73 genome sequence as a reference (MaizeGDB)^2^. All the amplified sequences were aligned using MEGA version 5^1^. Nucleotide polymorphisms, including SNPs and InDels, were identified (MAF ≥ 0.05). The significance of each DNA polymorphism associated with maize salt tolerance was calculated by Tassel 3.0 (Bradbury et al., 2007).

### Expression Analysis of *ZmHKT1;5*

Leaf samples for gene expression analyses were collected after 7 days of salt treatment. The responsiveness of *ZmHKT1;5* gene expression to salt stress was analyzed by qRT-PCR. Total RNA was isolated using RNAiso Plus reagent (TaKaRa) from no less than 3 seedlings. To remove genomic DNA contamination, total RNA was treated with the TURBO DNA-free^TM^ Kit (Ambion). The concentration of total RNA was determined using a Nanodrop2000c (Thermo Scientific, United States). One microgram of total RNA was used to synthesize cDNA with the TransScript All-in-One First-Strand cDNA Synthesis SuperMix for qPCR kit (Transgen Biotech). *Actin* was used as an endogenous standard to normalize the expression data of *ZmHKT1;5*. The primers used for quantitative PCR amplification were actin Fw: 5′-CTGAGGTTCTATTCCAGCCATCC, actin Rev: 5′-CCACCACTGAGGACAACATTACC, qHKT Fw: 5′-TCAACTTCAGCGTCCTCAACA, and qHKT Rev: 5′-GAATCCCACGTTGCCATACG. The SYBR Prime Script RT-PCR Kit (TaKaRa) was used for quantitative RT-PCR. Three replications were performed for each sample. Data were quantified using the comparative CT method (2^−ΔΔCT^ method) (Livak and Schmittgen, 2001).

### Tobacco Transformation

The coding region of the *ZmHKT1;5* cDNA was synthesized in accordance with the sequence of the maize B73 inbred line (1404 bp) and named as *ZmHKT1;5 AA*, the other *ZmHKT1;5* cDNA with two substituted sites, A134G and A433G (the location of the initiation codon (ATG) is marked as + 1) according to the association analysis results, was named as *ZmHKT1;5 GG*. The synthesized sequences were inserted into the *pCAMBIA3301* vector and driven by 35S promoter. And then the constructed vectors and empty vector were transformed into *Agrobacterium tumefaciens*, respectively. Tobacco (*Nicotiana tabacum* cv. Petite Havana SRI) was transformed as described (Wei et al., 2016). Using phosphinothricin selection, we obtained several independent T3 transgenic lines, and the expression of *ZmHKT1;5* was confirmed by RT-PCR in these lines.

### Salt Tolerance Assay in Transgenic Plants

To compare the germination rate between the vector control (VC) and the transgenic under salt stress, seeds were surface-sterilized with 70% alcohol for 1 min and 3% NaClO (v/v) for 10 min, washed at least three times with sterile water, and germinated on 1/2 MS + 100 mM NaCl medium supplemented with 1% sucrose and 0.8% agar. Three replications were performed.

To assess the salt tolerance of transgenic plants in different growth stages, the seeds were sowed in pots filled with enriched soil and cultured at 22°C and 70% relative humidity under a day/night cycle of 16 h/8 h using artificial light. Fifteen-day-old seedlings were watered with 100 mM NaCl solution every 2 days. The salt treatment was sustained for 15 days, and then the survival rate was calculated. Fifty-day-old seedlings were treated with 200 mM NaCl solution. After 3 days of treatment, the phenotype data was recorded. Three replications were performed.

Malondialdehyde (MDA) and H_2_O_2_ content and the activities of peroxidase (POD), superoxide dismutase (SOD), and catalase (CAT) in leaves were assayed using detection kits (MDA-1-Y, H_2_O_2_-1-Y, POD-1-Y, SOD-1-Y, and CAT-1-Y) from Suzhou Comin Biotechnology Co. Ltd., following the manufacturer’s instructions. Meanwhile, we also detected the Na^+^ and K^+^ contents of shoots by atomic absorption spectrophotometry (Lin et al., 2004). At least 30 plants of each line were compared with VC in each test, and statistical data were obtained from three independent experiments. All values were the means of three assays carried out for each value. Data considered significant at *p*-values < 0.05.

## Results

### Identification of *ZmHKT* Genes in Maize

**FIGURE 1:**
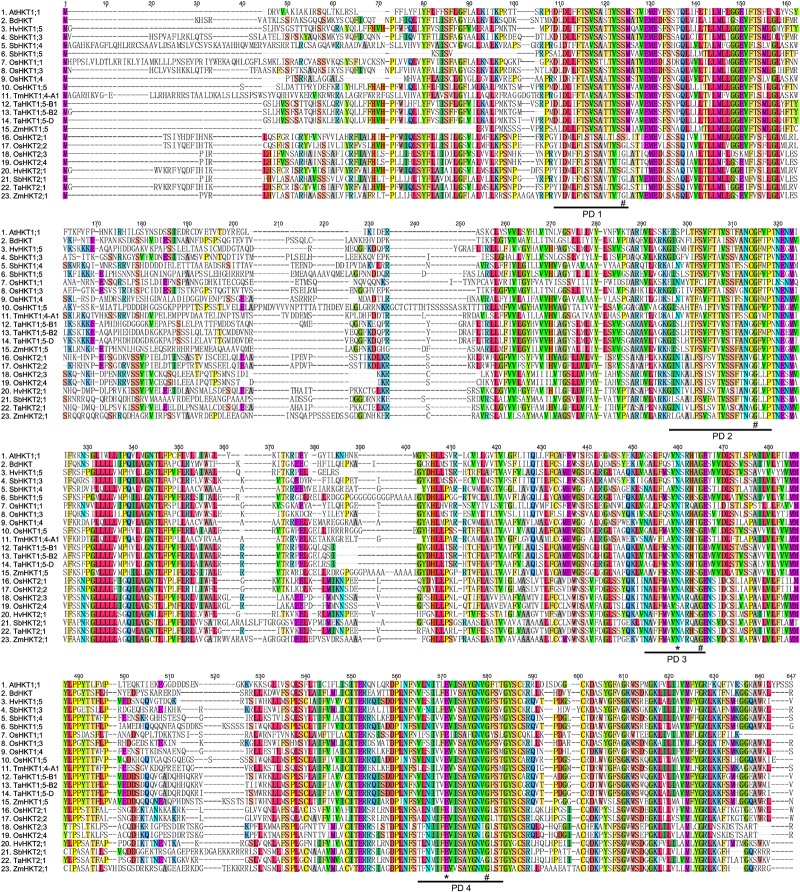
Characteristics of high-affinity potassium transporters in maize. A sequence alignment of the four-putative selectivity pore-forming regions (PD1 to PD4) of HKT proteins in higher plants. “#” indicates amino acid positions where glycine and serine residues are conserved. Amino acids marked with ^∗^ are known to confer salt tolerance in plants.

We used the Arabidopsis *AtHKT1;1* (*AtHKT1*) amino acid sequence to identify orthologues of *HKT* genes in the B73 maize genome database. Two highly orthologous sequences, NM_001175105 and XM_008646809, were discovered. According to the community nomenclature standard and the alignment results of plant HKT proteins (Platten et al., 2006), these two genes were named *ZmHKT1;5* (NM_001175105) and *ZmHKT2;1* (XM_008646809), respectively. The full lengths were 1,404 bp for *ZmHKT1;5* and 1,668 bp for *ZmHKT2;1*. The translated amino acid sequences were highly homologous to the corresponding sorghum proteins, SbHKT1;5 and SbHKT2;1, and the similarity was 89% and 82%, respectively (Wang et al., 2014). **Figure 1** shows other plant HKT-type proteins aligned with the two ZmHKT proteins. All maize HKT transporters contained four membrane-pore-membrane (MPM) motifs as same as other known plant HKT proteins. The key serine or glycine residues in maize HKT proteins were also highly conserved (marked with # in **Figure 1**).

**FIGURE 2:**
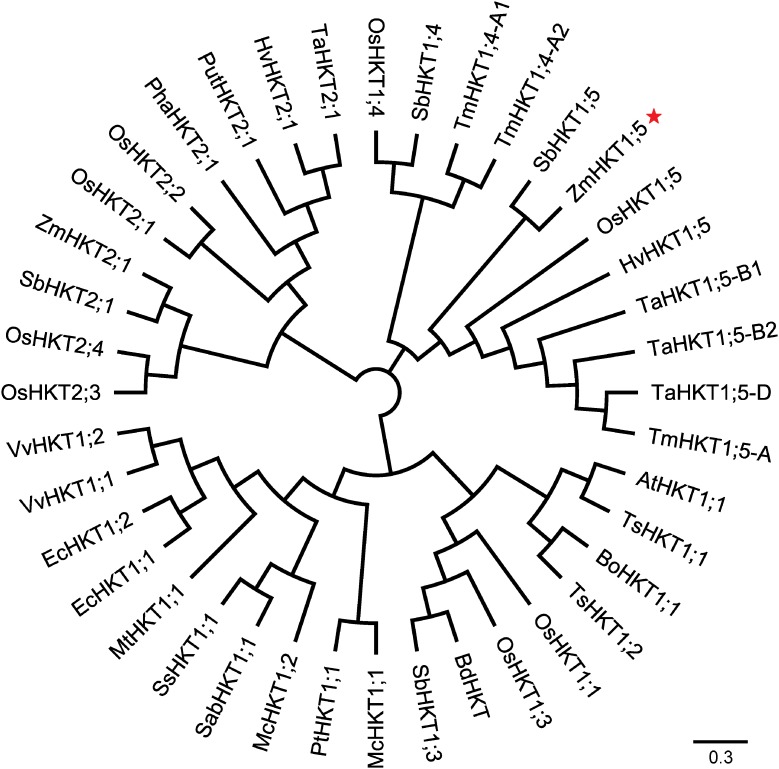
An unrooted phylogenetic tree of protein sequences of HKT homologs. Accession numbers for gene names are listed in **Supplementary Table S1**.

To understand more about the phylogenetic relationships of maize HKT proteins to orthologues in other species, standard protein-protein BLAST searches in the GenBank and Phytozome databases were carried out, and 40 entries were retrieved (cut-off *E*-value at 0.001) for HKT-like proteins from plants. Using these publicly available HKT proteins, a phylogenetic tree was constructed. And the results showed that there were two major clades in the HKT families (**Figure 2**). Depending on the key amino acid residues positioned at the first P-Loop region, ZmHKT1;5 belongs to Subfamily 1, with a serine residue in the first filter at the 32nd site, whereas ZmHKT2;1 belongs to Subfamily 2, with a glycine residue at the 83rd site (Maser et al., 2002). Furthermore, ZmHKT1;5 is highly conserved in the two amino acid residues (marked with ^∗^ in **Figure 1**) which are the key residues to effect the salt-tolerant trait in plants (Diatloff et al., 1998; Rubio et al., 1999). Thus, this study is focused on the characterization of ZmHKT1;5, and further research on ZmHKT2;1 will be reported elsewhere.

### Association Analysis of *ZmHKT1;5* Natural Variation With Salt Tolerance

**FIGURE 3:**
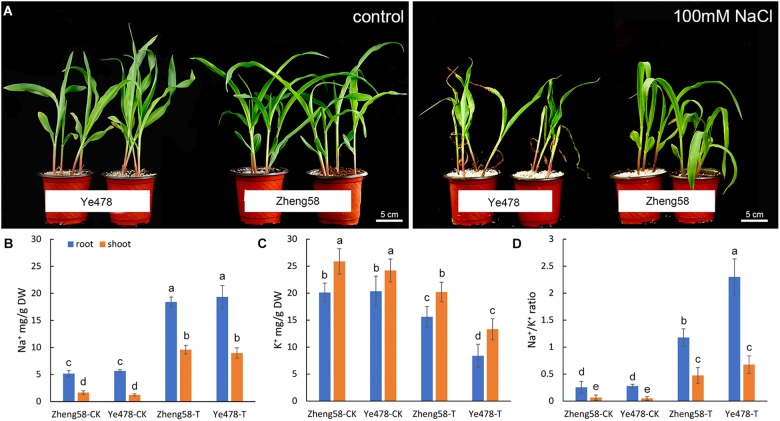
Salinity effects on salt-tolerant and salt-sensitive maize inbred lines. **(A)** Effects of 100mM NaCl applied for 7 days on the growth and development of two maize inbred lines with contrasting salt tolerance (Zheng58, tolerant; Ye478, sensitive). **(B,C)** Na^+^ and K^+^ content and Na^+^/K^+^ ratio illustrated in inbred lines (Zheng58, tolerant; Ye478, sensitive). “T” indicates 100mM NaCl treatment. After 3 days, seedling shoots were harvested and their ion content was measured by inductive coupled plasma emission spectrometry. The mean values ± SD for Na^+^ and K^+^ content and for Na^+^/K^+^ ratio **(D)**, calculated from the data in **(B**,**C)**, are illustrated. Data from three experiments with similar results are shown. Columns with different capital letters indicate significant differences at *P* < 0.05 (Duncan test).

A collection of 54 maize inbred lines from diverse geographical origins were investigated to assess the genetic diversity associated with salt tolerance. We selected the leaf relative water content as a main parameter, and the phenotype variation was also recorded following 7 days of 100 mM NaCl salt stress (**Supplementary Table S1**). Two of the inbred lines, Zheng58 and Ye478, showed the most extreme phenotypes in the assay (Zheng58: tolerant; Ye478: sensitive), and were used to analyze Na^+^/K^+^ ratio. The results showed that the Na^+^/K^+^ ratio of Zheng58 grew much better than Ye478 after the salt treatment, although neither of these two lines grew well like the control (**Figure 3A**).

To a large extent, the growth difference between Zheng58 and Ye478 was attributable to the Na^+^/K^+^ ratio in plant tissues. Na^+^ content in shoots and roots of control plants was in the range of 3–6 mg/g and was not obviously different between the two inbred lines. However, a salt treatment resulted in a distinctly Na^+^ accumulation and K^+^ reduction. Compared with Zheng58, Ye478 was similar to Zheng58 in the accumulation of Na^+^ in both the shoots and roots, but the more K^+^ decrease was detected in the shoots and roots (**Figures 3B,C**). A lower Na^+^/K^+^ ratio was consequently observed in the salt-tolerant inbred line, Zheng58 (*P* < 0.05) (**Figure 3D**). These results implied that the salt-tolerant line, Zheng58, maintained a better Na^+^/K^+^ balance in tissues than the salt-sensitive one, Ye478. Under the salt stress, maintaining a proper Na^+^/K^+^ ratio, especially in roots, may be an important strategy for improving the maize salt-tolerance.

To identify the DNA polymorphisms of *ZmHKT1;5* in maize, we sequenced the *ZmHKT1;5* gene in the 54 maize inbred lines. A 2.5 kb genomic DNA fragment was amplified and sequenced, which included the *ZmHKT1;5* coding region and both the 5′ and 3′ UTRs. Totally, 61 SNPs and 12 insertions or deletions (InDels) were identified in the entire sequence of *ZmHKT1;5*. In the coding region, none of the amino acid alterations were observed in the conserved regions that were identified by Uozumi et al. (2000), and also no changes were detected at the first serine residue of PD1 that has been involved in the Na^+^ specificity (Maser et al., 2002).

**FIGURE 4:**
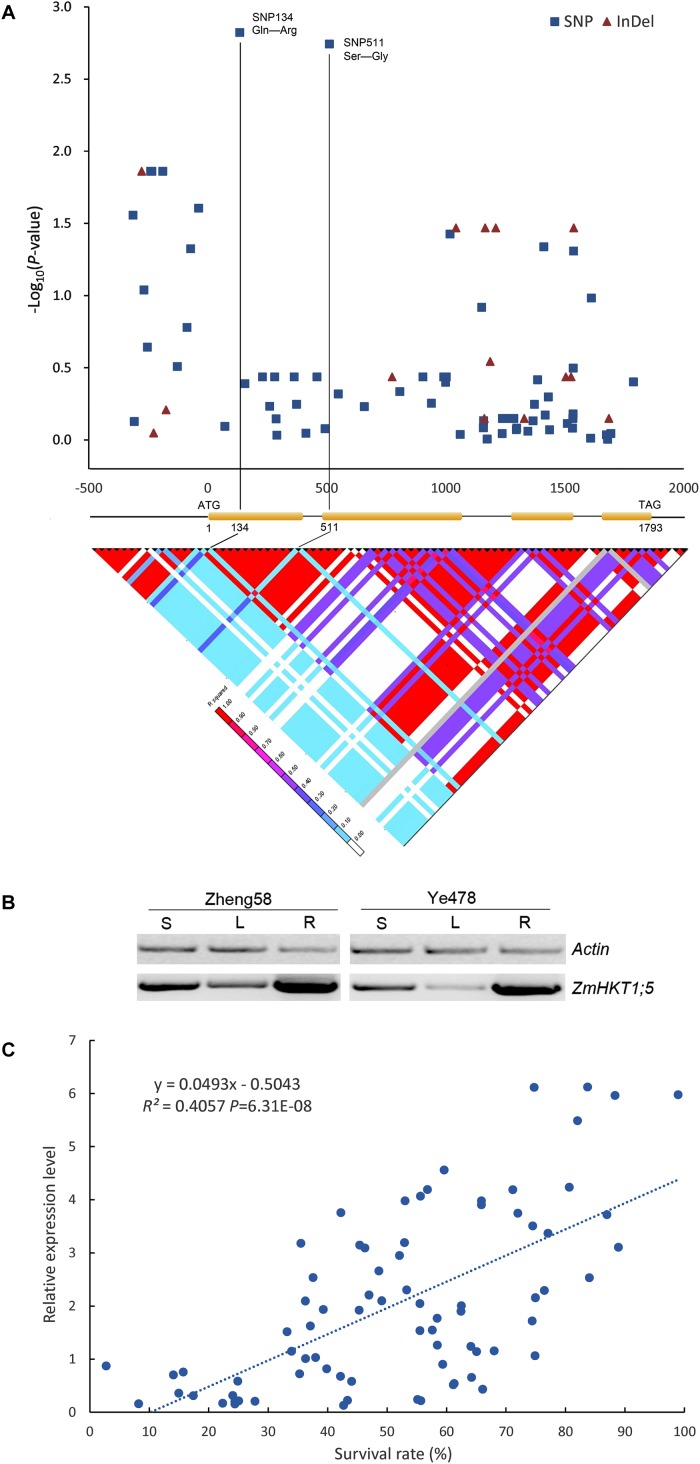
Association analysis of genetic variation in *ZmHKT1;5* with salt tolerance. **(A)** Association analysis of genetic variation *ZmHKT1;5* with maize salt tolerance and the pattern of pairwise LD of DNA polymorphisms in the *ZmHTK1;5* gene. A schematic diagram of the 2.5 kb genomic fragment, including the 5′ and 3′ UTRs, four exons and three introns is presented. The location of the start codon is labeled “1”. The *P*-value is shown on a −log_10_ scale. The two most significant non-synonymous variations in the coding region are connected to their locations in the gene diagram by solid lines. SNP 134 produced a change of Pro to Gln in the encoded protein, and SNP 511 changed Ser to Gly. **(B)** RT-PCR analysis of the expression levels of *ZmHKT1;5* in Zheng58 (salt-tolerant) and Ye478 (salt-sensitive). RNA was extracted from the leaf sheath (S), leaf blade (L), and root (R) of seedlings. **(C)** Correlation analysis of survival rate with the relative expression level of *ZmHKT1;5.* Salt stress was applied to the maize seedlings under 100mM NaCl conditions.

Results indicated that two newly identified polymorphisms (SNPs 134 and 511), which were located downstream of the start codon (ATG site) of ZmHKT1;5, were in strong LD (*r*^2^ ≥ 0.8) and were significantly associated with phenotypic variation under salt stress (*P* = 1.50 × 10-3 and *P* = 1.81 × 10-3, respectively), contributing 5.01% of the phenotypic variation in the natural population. Additionally, the two SNPs (SNPs 134 and 511) are non-synonymous; SNP 134 resulted in an amino acid residue change of Glutamine (Gln, CAG) to Arginine (Arg, CGG), and SNP511 resulted in an alteration of Serine (Ser, AGC) to Glycine (Gly, GGC) (**Figure 4A** and **Supplementary Figure S1**).

According to the topological model of the Arabidopsis HKT, ZmHKT1;5 protein may also contain eight similar transmembrane domains (TMDs) (Kato et al., 2001). One variation (SNP A134G, amino-acid: Q45R) in ZmHKT1;5 lay in the loop between TMD1 and TMD2, and the other (SNP A511G, amino-acid: S145G) was located in the loop between TMD2 and TMD3 (**Supplementary Figure S1a**). These two amino-acid substitutions may be associated with the functional differences of HKT1;5 in different maize inbred lines.

To determine whether differences in gene expression contributed to salt tolerance, we examined the expression patterns of *ZmHKT1;5* in the two inbred lines (Zheng58 and Ye478), firstly. As shown in **Figure 4B**, the expression of *ZmHKT1;5* was relatively more abundant in the root, sheath, and leaf of the salt-tolerant inbred line Zheng58. In addition, the mRNA levels of *ZmHKT1;5* under salt stress were quantified in all 54 lines. Results showed that *ZmHKT1;5* expression was positively correlated with increased survival rate under salt stress (100 mM NaCl for 7 days) (**Figure 4C**). The results demonstrated that *ZmHKT1;5* expression level was important for the survival of maize seedlings under salt stress.

### Overexpression of *ZmHKT1;5* Enhances Salt Tolerance in Transgenic Tobacco Plants

**FIGURE 5:**
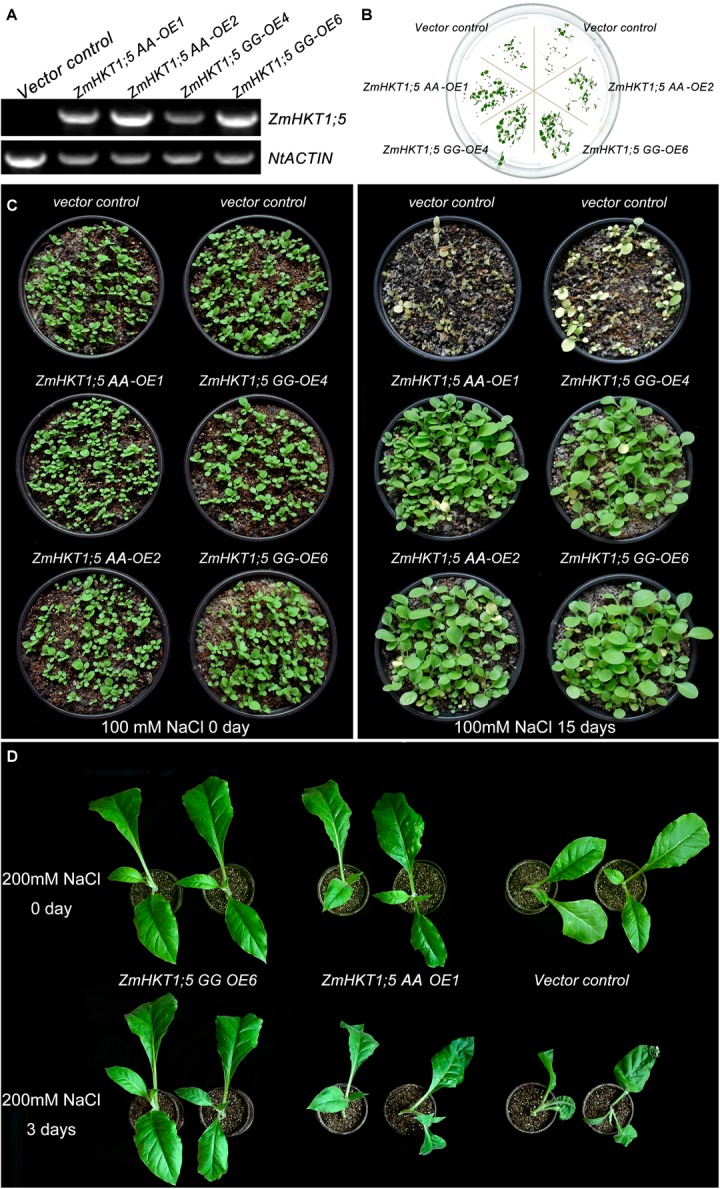
Phenotypes of transgenic tobacco under salt stress. **(A)** Expression of *ZmHKT1;5* in the transgenic lines. **(B)** Seed germination in VC and transgenic plants under 100mM NaCl conditions. **(C)** Phenotypes of 15-day-old VC and transgenic seedlings before (left) and after (right) treatment with 150 mM NaCl for 15 days. **(D)** Phenotypes of 50-day-old VC and transgenic plants after treatment with 200 mM NaCl for 3 days.

Because *ZmHKT1;5* was upregulated by NaCl treatment, transgenic tobacco plants expressing *ZmHKT1;5* were generated to examine the role of *ZmHKT1;5* in salt stress response. In total, four putative *ZmHKT1;5* overexpressing lines were confirmed by PCR. Results showed that *ZmHKT1;5* was detected in transgenic plants but not in VC. *NtActin* was used as an endogenous control for RT-PCR analysis (**Figure 5A**). T3 lines (*ZmHKT1;5 AA-OE1*, *ZmHKT1;5 AA-OE2*, *ZmHKT1;5 GG-OE4*, and *ZmHKT1;5 GG-OE6*) had a nearly 100% germination rate on MS medium containing 100 mg/L of kanamycin and were therefore thought to be homozygous transgenic lines for further analysis.

To test the effects of *ZmHKT1;5* overexpression in the response to salt stress, the germination of *ZmHKT1;5*-overexpressing seeds was firstly examined. The seed-germinating rate was no obvious difference between VC and transgenic plants under the normal condition. Under the 100 mM NaCl condition, 82.43% of the *ZmHKT1;5*-overexpressing seeds germinated in comparison with 13.29% of VC seeds. In addition, the germination rate of the two *ZmHKT1;5 GG-*overexpressing lines was obviously higher than that of the two *ZmHKT1;5 WT*-overexpressing lines (**Figure 5B**).

To test the effect of *ZmHKT1;5* overexpression on salt tolerance in the seedling stage, 15-day-old T3 homozygous transgenic and VC seedlings were treated with 100 mM NaCl. After 15 days, all VC seedlings had died, but most of the *ZmHKT1;5* overexpressing seedlings were still growing and appeared healthy (**Figure 5C**). Then, fifty-day-old transgenic and VC seedlings were watered with 200 mM NaCl for 3 days to compare the difference in salt tolerance between transgenic and VC seedlings. The results showed that slight dehydration was observed in *ZmHKT1;5 AA OE-1* transgenic seedlings and severe dehydration was observed in VC seedlings, by contrast, no obvious phenotypic changes showed in the *ZmHKT1;5 GG OE-6* transgenic seedlings (**Figure 5D**). These results indicated that the overexpression of *ZmHKT1;5* improved salt tolerance in transgenic tobacco, and overexpressing *ZmHKT1;5 GG* performed better than overexpressing *ZmHKT1;5 AA.*

### Overexpression of *ZmHKT1;5* Decreases MDA and Na^+^/K^+^ Ratio Under Salt Stress

**FIGURE 6:**
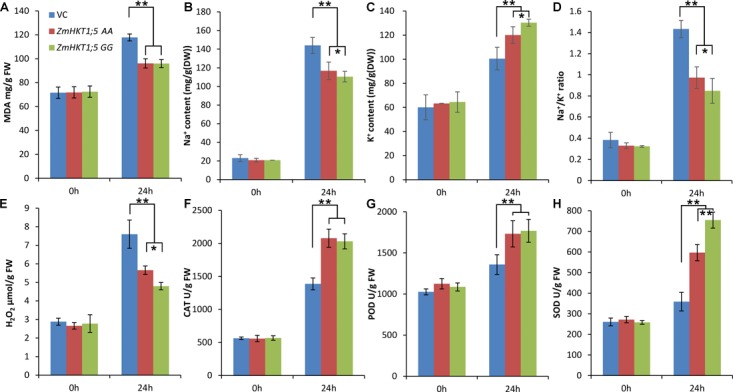
Analysis of MDA content, Na^+^/K^+^ ratio, three antioxidant enzyme activities and H_2_O_2_ accumulation in VC and transgenic plants under salt stress. 30-day-old seedlings were treated with 150mM NaCl for 72 h, then the leaves were sampled to assess the MDA content **(A)**, Na^+^ content **(B)**, K^+^ content **(C)**, Na^+^/K^+^ ratio **(D)**, H_2_O_2_ content **(E)**, CAT activity **(F)**, POD activity **(G)**, and SOD activity **(H)**. Data are the means ± SD calculated from three replicates. ^∗^ and ^∗∗^ indicate that the value in transgenic plants are significantly different (*P* < 0.05) and extremely significantly different (*P* < 0.01) from that of VC plants, respectively. Three biological replications produced similar results.

These indications that the overexpression of *ZmHKT1;5* enhanced salt tolerance led us to detect the changes of the physiological status induced by *ZmHKT1;5* overexpressing. Thirty-day-old T3 homozygous transgenic plants were treated with 150 mM NaCl for 24 h. Following the treatment, MDA content in VC plants increased up to 117.8 mg/g, whereas only 96.07 mg/g and 95.96 mg/g was observed in the *ZmHKT1;5 AA* and *ZmHKT1;5 GG* transgenic plants, respectively. The MDA content of the transgenic plants was significantly lower than that of VC plants (**Figure 6A**), indicating that VC plants were subjected to more severe oxidative membrane damage after salt stress. The capacity of plants to maintain a low Na^+^/K^+^ ratio is another key role of plant salt tolerance. In this study, the Na^+^/K^+^ ratio was also obviously lower in the transgenic plants (**Figure 6B**). These results indicated that *ZmHKT1;5* overexpressing lines possessed more powerful resistance to salt stress. It is noteworthy that the Na^+^/K^+^ ratio of overexpressing *ZmHKT1;5 GG* plants was significantly lower than that of overexpressing *ZmHKT1;5 AA* plants. This result indicated that two SNP might affect the ability of ZmHKT protein to transport Na^+^.

### Overexpression of *ZmHKT1;5* Increases Antioxidant Enzyme Activity and Decreases H_2_O_2_ Content Under Salt Stress

Because enzymatic antioxidants can affect cellular reactive oxygen species (ROS) levels, we also detected the activity of SOD, POD and CAT in the leaves of VC and transgenic plants to further analyze the relationships between enzymatic antioxidants and the influence of *ZmHKT1;5* overexpression on salt-stress tolerance. The results showed that after salt treatment, the SOD, POD, and CAT activities in transgenic plants were significantly higher than those in VC plants; meanwhile, the H_2_O_2_ levels were lower in the transgenic plants (**Figures 6C–F**). These results indicated that overexpression of *ZmHKT1;5* reduced ROS levels by increasing the activity of some antioxidant enzymes (e.g., SOD, POD, and CAT) under salt stress. We also found SOD activity in overexpressing *ZmHKT1;5 GG* plants was significantly higher than those in overexpressing *ZmHKT1;5 AA* plants, while the same difference was not detected in CAT and POD analysis. The difference of SOD activity might be a reason for the obvious difference of H_2_O_2_ levels in the two kinds of *ZmHKT1;5* overexpressing plants.

## Discussion

Agricultural production is seriously threatened by increasing soil salinity. Some Na^+^ transporters in plants play an important role in the response to salt stress. Amongst all Na^+^ transporters, HKT has been demonstrated to have a vital role in many species of plants in the salt-tolerance.

In this study, we examined the correlation between *HKT* genes and salt tolerance in maize. Two *HKT* gene family members were identified, and we just focused on the analysis of *ZmHKT1;5* in this study. A range of heterogeneous expression experiments suggested that *ZmHKT1;5* had a remarkable ability to maintain optimal Na^+^/K^+^ balance and plant growth under the salt stress, which is similar to the effects of HKT genes normally identified in some halophytic plants.

*HKT* genes have been given a nomenclature according to biophysical transport and phylogenetic analyses and divided into two groups based on amino acid sequence (Maser et al., 2002; Platten et al., 2006). A Gly/Ser residue located in the first pore loop differs between group 1 and group 2 HKTs. A Ser could be found in all Group 1 HKT transporters; this may make them more selective for Na ^+^ (Uozumi et al., 2000; Horie et al., 2001; Maser et al., 2002; Garciadeblas et al., 2003; Ren et al., 2005; Sunarpi et al., 2005; Platten et al., 2006). Most of Group 2 HKT transporters show a larger K^+^ permeability and Na^+^ permeability (Horie et al., 2001, 2009; Golldack et al., 2002; Haro et al., 2005; Almeida et al., 2013). The phylogenetic analysis placed the maize *HKT* sequences amongst the sequences of Arabidopsis, cereals and halophytes. In addition, the results of the multiple sequence alignment showed that the ZmHKT1;5 protein shares the Group 1 similarity with HKT1;5 proteins such as OsHKT1;5, which encodes a member of the HKT1 subfamily in the *Trk/Ktr/HKT* transporter family, suggesting that *ZmHKT1;5* may share a similar function with *OsHKT1;5*. *OsHKT1;5* is expressed in rice xylem parenchyma cells and reduces Na^+^ levels in the xylem sap; in addition, *OsHKT1;5* encodes a Na^+^-selective transporter and can exclude Na^+^ from shoots under the salt stress protecting leaves from Na^+^ toxicity (Ren et al., 2005; Sunarpi et al., 2005; Rus et al., 2006). The Na^+^/K^+^ ratio was detected in the transgenic tobacco study, which showed that the *ZmHKT1;5*-overexpressing plants had higher K^+^ content and a better-balanced Na^+^/K^+^ ratio to reduce salt-stress symptoms. Recently, some HKT genes have been identified in sorghum and were also shown to contribute to salt tolerance by enabling K^+^ uptake under salt stress (Oomen et al., 2012; Wang et al., 2014). All these studies indicated that HKTs in glycophytic crops could play a similar role to those in halophytes.

Natural variations or alleles of genetic loci are believed to be useful in breeding. Recently, candidate gene association analysis has been developed to dissect natural variations or alleles of certain genes contributing to complex traits. Using this method, researchers have successfully identified favorable variations/alleles of *ZmDREB2.7*, *ZmNAC111*, *ZmPP2C-A*, and *ZmVPP1* for enhancing maize abiotic stress tolerance (Liu et al., 2013; Mao et al., 2015; Wang et al., 2016; Xiang et al., 2017). In this study, we detected favorable variations/alleles (SNPs A134G and A511G) of *ZmHKT1;5* in the same method. These two SNPs does not change the amino acid residues that are known to confer salt tolerance in wheat (Rubio et al., 1995, 1999). Thus, the affinity to Na^+^ of ZmHKT1;5 is probably conferred by these conserved amino acid residues. However, further experiments are required to validate the effect of these two SNPs on K^+^ uptake. Moreover, it is noteworthy that polymorphisms of *cis*-regulators was significantly correlated with HKT expression and salt tolerance in some genome wide association studies of Arabidopsis (Baxter et al., 2010; Julkowska et al., 2017). In our study, we also detected associations with *ZmHKT1;5* in the promoter region, but no significant correlation sites were identified (data not showed). The results showed that SNP in the coding region of *ZmHKT1;5* could affect the function of genes which was similar to those of *OsGA2ox6* and *GA2ox9A* (Lo et al., 2017; Ford et al., 2018). Although it is still unclear how the SNPs 134G and 511G of *ZmHKT1;5* leads to functional differences, they should be favorable because the most of the salt-tolerant maize inbred lines tended to contain this allele, while the most of the salt-susceptible lines tended to have SNP 134A and 511A. SNPs A134G, and A511G could be used in breeding programs as candidate markers to screen salt-tolerant lines in maize.

To understand the role of *ZmHKT1;5* in plant salt tolerance further, *ZmHKT1;5* was transformed into tobacco in the forms of gene sequence with SNPs 134A and 511A (*ZmHKT1;5 AA*) and with SNPs 134G and 511G (*ZmHKT1;5 GG*). Under normal conditions, *ZmHKT1;5* transgenic plants were similar to VC plants in terms of germination, biomass and antioxidant enzyme activities. However, when exposed to NaCl stress, the transgenic tobacco seedlings displayed better performance in many respects than VC seedlings, including higher germination rate as well as SOD, CAT and POD activities. In addition, Na^+^/K^+^ ratio, MDA content, and H_2_O_2_ accumulation in the transgenic plants were less than those in VC plants. Especially, the overexpressing *ZmHKT1;5 GG* plants were more tolerant than overexpressing *ZmHKT1;5 AA* under salt stress. These results implied that overexpression of *ZmHKT1;5* could effectively improve plant salt tolerance and SNPs A134G and A511G of *ZmHKT1;5* might be the important candidate sites to affect the function of ZmHKT protein. The mechanism should be studied in the future.

Abiotic stress always damages cell membrane systems, inhibits photosynthesis, and limits plant productivity, which is due to accumulate more ROS (Negi et al., 2015). In this study, *ZmHKT1;5* overexpression plants showed less ROS accumulation when they were exposed to salt stress (**Figure 6**), suggesting that the regulation of salt response processes by *ZmHKT1;5* involved ROS metabolism, similar to other stress response-related genes. One hypothesis to explain Na^+^ level changes in plants is that ROS might stimulate *HKT* expression or activity. Alterations in ROS levels in plants may be a crucial determinant of transporter activity (Almeida et al., 2013). According to our results, this hypothesis could be improved. First, ROS accumulation might be induced under salt stress. Next, ROS would activate the expression of *ZmHKT1;5*. ZmHKT1;5 could regulate the Na^+^/K^+^ ratio to repress ROS accumulation and preserve plant growth under salt stress. The regulation pathway by which ROS activates the expression of *ZmHKT1;5* should be identified in the further studies. Some studies have found that *DREB* transcript levels were coupled with the accumulation of ROS under salt stress (Yan et al., 2014; Yang et al., 2017), indicating the possible regulation of *DREB* by ROS accumulation. Therefore, transcription factors such as *DREB* and *WRKY* represent possible candidate genes for involvement in this process.

## Conclusion

HKTs are important salt-stress response-related proteins, and some studies of HKT involvement in salt stress tolerance have been reported in glycophytic plants. However, there have been few studies on stress responses involving *HKT* in maize. In the current study, an *HKT* gene was cloned from maize and named *ZmHKT1;5*. *ZmHKT1;5* belongs to the *HKT1* subfamily. Two SNPs of *ZmHKT1;5* were significantly associated with phenotypic variation in salt tolerance and thus represent candidate markers for screening salt-tolerant maize lines. A rapid induction of *ZmHKT1;5* gene expression in response to salt stress was important in enhancing plant salt-stress tolerance. Overexpressing *ZmHKT1;5* can improve salt tolerance in transgenic plants. The *ZmHKT1;5* overexpression tobacco seedlings showed enhanced germination rate as well as CAT, SOD, and POD activities in comparison with VC seedlings. The content of MDA and H_2_O_2_ that were generated in VC plants were all greater than they were in *ZmHKT1;5* overexpression plants when exposed to salt stress, which indicated that *ZmHKT1;5* overexpression improved plant tolerance to NaCl. These results suggested that *ZmHKT1;5* should be an excellent candidate gene for molecular breeding to improve plant stress tolerance. Especially, the two SNPs (A134G and A511G of *ZmHKT1;5*) significantly affected the salt tolerance of transgenic tobacco seedlings, which might be two important structural sites related to ZmHKT1;5 function. Further studies should concentrate on the effects of the two sites on ZmHKT1;5 structure and function.

## Author Contributions

YiL and XS conceived and designed the experiments. ZJ and GS assembled sequences of HKT genes and performed the phylogenetic analyses. ZW and YaL performed the tobacco transformation. CJ performed the expression analysis of the *ZmHKT1;5* Gene. YJ and FJ carried out the salt tolerance assay, data collection and analysis. YiL and XS drafted the manuscript and generated the figures and tables. All the authors agreed on the contents of the paper.

## Conflict of Interest Statement

The authors declare that the research was conducted in the absence of any commercial or financial relationships that could be construed as a potential conflict of interest.

## Footnotes

1 http://www.megasoftware.net/

2 http://www.maizegdb.org/

## Supplementary Material

FIGURE S1 Structural model of ZmHKT1;5. **(a)** A model of the ZmHKT1;5 protein based on hydrophobicity plot analysis and the eight-TMD model reported previously (Kato et al., 2001). Asterisks indicate positions of residue substitution. **(b)** A protein structure diagram of ZmHKT1;5. SNP A134G and SNP A511G are marked.

TABLE S1 Names and accession numbers of proteins used for phylogenetic analysis.

TABLE S2 Tolerance to salt stress of different inbred lines.

## Abbreviations

CAT: catalase
HKT: High-affinity potassium transporter
MDA: malondialdehyde
POD: peroxidase
ROS: reactive oxygen species
SOD: superoxide dismutase
VC: vector control.

## References

[B1] Almeida P. Katschnig D. De Boer A. H. (2013). HKT transporters–state of the art. *Int. J. Mol. Sci.* 14 20359–20385. 10.3390/ijms141020359 24129173 PMC3821619

[B2] Baxter I. Brazelton J. N. Yu D. Huang Y. S. Lahner B. Yakubova E. (2010). A coastal cline in sodium accumulation in *Arabidopsis thaliana* is driven by natural variation of the sodium transporter AtHKT1;1. *PLoS Genet.* 6:e1001193. 10.1371/journal.pgen.1001193 21085628 PMC2978683

[B3] Bradbury P. J. Zhang Z. Kroon D. E. Casstevens T. M. Ramdoss Y. Buckler E. S. (2007). TASSEL: software for association mapping of complex traits in diverse samples. *Bioinformatics* 23 2633–2635. 10.1093/bioinformatics/btm308 17586829

[B4] Corratge-Faillie C. Jabnoune M. Zimmermann S. Very A. A. Fizames C. Sentenac H. (2010). Potassium and sodium transport in non-animal cells: the Trk/Ktr/HKT transporter family. *Cell. Mol. Life. Sci.* 67 2511–2532. 10.1007/s00018-010-0317-7 20333436 PMC11115768

[B5] Cramer G. R. (1992). Kinetics of maize leaf elongation: III. Silver thiosulfate increases the yield threshold of salt-stressed plants, but ethylene is not involved. *Plant Physiol.* 100 1044–1047. 10.1104/pp.100.2.1044 16653015 PMC1075664

[B6] Diatloff E. Kumar R. Schachtman D. P. (1998). Site directed mutagenesis reduces the Na^+^ affinity of HKT1, an Na^+^ energized high affinity K^+^ transporter. *FEBS Lett.* 432 31–36. 10.1016/S0014-5793(98)00833-3 9710245

[B7] Ford B. Foo E. Sharwood R. E. Karafiatova M. Vrana J. Macmillan C. (2018). Rht18 semi-dwarfism in wheat is due to increased expression of GA 2-oxidaseA9 and lower GA content. *Plant Physiol.* 177 168–180. 10.1104/pp.18.00023 29545269 PMC5933146

[B8] Garciadeblas B. Senn M. E. Banuelos M. A. Rodriguez-Navarro A. (2003). Sodium transport and HKT transporters: the rice model. *Plant J.* 34 788–801. 10.1046/j.1365-313X.2003.01764.x 12795699

[B9] Geilfus C. M. Zorb C. Muhling K. H. (2010). Salt stress differentially affects growth-mediating beta-expansins in resistant and sensitive maize (*Zea mays* L.). *Plant Physiol. Biochem.* 48 993–998. 10.1016/j.plaphy.2010.09.011 20970350

[B10] Golldack D. Su H. Quigley F. Kamasani U. R. Munoz-Garay C. Balderas E. (2002). Characterization of a HKT-type transporter in rice as a general alkali cation transporter. *Plant J.* 31 529–542. 10.1046/j.1365-313X.2002.01374.x 12182709

[B11] Haro R. Banuelos M. A. Rodriguez-Navarro A. (2010). High-affinity sodium uptake in land plants. *Plant Cell Physiol.* 51 68–79. 10.1093/pcp/pcp168 19939835

[B12] Haro R. Banuelos M. A. Senn M. E. Barrero-Gil J. Rodriguez-Navarro A. (2005). HKT1 mediates sodium uniport in roots. Pitfalls in the expression of HKT1 in yeast. *Plant Physiol.* 139 1495–1506. 10.1104/pp.105.067553 16258014 PMC1283784

[B13] Horie T. Hauser F. Schroeder J. I. (2009). HKT transporter-mediated salinity resistance mechanisms in Arabidopsis and monocot crop plants. *Trends Plant Sci.* 14 660–668. 10.1016/j.tplants.2009.08.009 19783197 PMC2787891

[B14] Horie T. Yoshida K. Nakayama H. Yamada K. Oiki S. Shinmyo A. (2001). Two types of HKT transporters with different properties of Na^+^ and K^+^ transport in *Oryza sativa*. *Plant J.* 27 129–138. 10.1046/j.1365-313x.2001.01077.x 11489190

[B15] Huang S. Spielmeyer W. Lagudah E. S. Munns R. (2008). Comparative mapping of HKT genes in wheat, barley, and rice, key determinants of Na^+^ transport, and salt tolerance. *J. Exp. Bot.* 59 927–937. 10.1093/jxb/ern033 18325922

[B16] James R. A. Blake C. Byrt C. S. Munns R. (2011). Major genes for Na^+^ exclusion, Nax1 and Nax2 (wheat HKT1;4 and HKT1;5), decrease Na^+^ accumulation in bread wheat leaves under saline and waterlogged conditions. *J. Exp. Bot.* 62 2939–2947. 10.1093/jxb/err003 21357768

[B17] Julkowska M. M. Koevoets I. T. Mol S. Hoefsloot H. Feron R. Tester M. A. (2017). Genetic components of root architecture remodeling in response to salt stress. *Plant Cell* 29 3198–3213. 10.1105/tpc.16.00680 29114015 PMC5757256

[B18] Kato Y. Sakaguchi M. Mori Y. Saito K. Nakamura T. Bakker E. P. (2001). Evidence in support of a four transmembrane-pore-transmembrane topology model for the *Arabidopsis thaliana* Na^+^/K^+^ translocating AtHKT1 protein, a member of the superfamily of K^+^ transporters. *Proc. Natl. Acad. Sci. U.S.A.* 98 6488–6493. 10.1073/pnas.101556598 11344270 PMC33495

[B19] Krill A. M. Kirst M. Kochian L. V. Buckler E. S. Hoekenga O. A. (2010). Association and linkage analysis of aluminum tolerance genes in maize. *PLoS One* 5:e9958. 10.1371/journal.pone.0009958 20376361 PMC2848604

[B20] Li L. Li H. Li Q. Yang X. Zheng D. Warburton M. (2011). An 11-bp insertion in *Zea mays fatb* reduces the palmitic acid content of fatty acids in maize grain. *PLoS One* 6:e24699. 10.1371/journal.pone.0024699 21931818 PMC3172307

[B21] Li Q. Li L. Yang X. Warburton M. L. Bai G. Dai J. (2010a). Relationship, evolutionary fate and function of two maize co-orthologs of rice GW2 associated with kernel size and weight. *BMC Plant Biol.* 10:143. 10.1186/1471-2229-10-143 20626916 PMC3017803

[B22] Li Q. Yang X. Bai G. Warburton M. L. Mahuku G. Gore M. (2010b). Cloning and characterization of a putative GS3 ortholog involved in maize kernel development. *Theor. Appl. Genet.* 120 753–763. 10.1007/s00122-009-1196-x 19898828

[B23] Lin H. X. Zhu M. Z. Yano M. Gao J. P. Liang Z. W. Su W. A. (2004). QTLs for Na^+^ and K^+^ uptake of the shoots and roots controlling rice salt tolerance. *Theor. Appl. Genet.* 108 253–260. 10.1007/s00122-003-1421-y 14513218

[B24] Liu S. Wang X. Wang H. Xin H. Yang X. Yan J. (2013). Genome-wide analysis of *ZmDREB* genes and their association with natural variation in drought tolerance at seedling stage of *Zea mays* L. *PLoS Genet.* 9:e1003790. 10.1371/journal.pgen.1003790 24086146 PMC3784558

[B25] Livak K. J. Schmittgen T. D. (2001). Analysis of relative gene expression data using real-time quantitative PCR and the 2^−ΔΔCT^ method. *Methods* 25 402–408. 10.1006/meth.2001.1262 11846609

[B26] Lo S. F. Ho T. D. Liu Y. L. Jiang M. J. Hsieh K. T. Chen K. T. (2017). Ectopic expression of specific GA2 oxidase mutants promotes yield and stress tolerance in rice. *Plant Biotechnol. J.* 15 850–864. 10.1111/pbi.12681 27998028 PMC5466439

[B27] Mao H. Wang H. Liu S. Li Z. Yang X. Yan J. (2015). A transposable element in a NAC gene is associated with drought tolerance in maize seedlings. *Nat. Commun.* 6:8326. 10.1038/ncomms9326 26387805 PMC4595727

[B28] Maser P. Hosoo Y. Goshima S. Horie T. Eckelman B. Yamada K. (2002). Glycine residues in potassium channel-like selectivity filters determine potassium selectivity in four-loop-per-subunit HKT transporters from plants. *Proc. Natl. Acad. Sci. U.S.A.* 99 6428–6433. 10.1073/pnas.082123799 11959905 PMC122965

[B29] Munns R. (2005). Genes and salt tolerance: bringing them together. *New Phytol.* 167 645–663. 10.1111/j.1469-8137.2005.01487.x 16101905

[B30] Munns R. Tester M. (2008). Mechanisms of salinity tolerance. *Annu. Rev. Plant Biol.* 59 651–681. 10.1146/annurev.arplant.59.032607.092911 18444910

[B31] Negi N. P. Shrivastava D. C. Sharma V. Sarin N. B. (2015). Overexpression of *CuZnSOD* from *Arachis hypogaea* alleviates salinity and drought stress in tobacco. *Plant Cell Rep.* 34 1109–1126. 10.1007/s00299-015-1770-4 25712013

[B32] Oomen R. J. Benito B. Sentenac H. Rodriguez-Navarro A. Talon M. Very A. A. (2012). HKT2;2/1, a K^+^ -permeable transporter identified in a salt-tolerant rice cultivar through surveys of natural genetic polymorphism. *Plant J.* 71 750–762. 10.1111/j.1365-313X.2012.05031.x 22530609

[B33] Platten J. D. Cotsaftis O. Berthomieu P. Bohnert H. Davenport R. J. Fairbairn D. J. (2006). Nomenclature for HKT transporters, key determinants of plant salinity tolerance. *Trends Plant Sci.* 11 372–374. 10.1016/j.tplants.2006.06.001 16809061

[B34] Qiu L. Wu D. Ali S. Cai S. Dai F. Jin X. (2011). Evaluation of salinity tolerance and analysis of allelic function of HvHKT1 and HvHKT2 in Tibetan wild barley. *Theor. Appl. Genet.* 122 695–703. 10.1007/s00122-010-1479-2 20981400

[B35] Ren Z. H. Gao J. P. Li L. G. Cai X. L. Huang W. Chao D. Y. (2005). A rice quantitative trait locus for salt tolerance encodes a sodium transporter. *Nat. Genet.* 37 1141–1146. 10.1038/ng1643 16155566

[B36] Riedelsheimer C. Czedik-Eysenberg A. Grieder C. Lisec J. Technow F. Sulpice R. (2012). Genomic and metabolic prediction of complex heterotic traits in hybrid maize. *Nat. Genet.* 44 217–220. 10.1038/ng.1033 22246502

[B37] Rodriguez-Navarro A. Rubio F. (2006). High-affinity potassium and sodium transport systems in plants. *J. Exp. Bot.* 57 1149–1160. 10.1093/jxb/erj068 16449373

[B38] Rubio F. Gassmann W. Schroeder J. I. (1995). Sodium-driven potassium uptake by the plant potassium transporter HKT1 and mutations conferring salt tolerance. *Science* 270 1660–1663. 10.1126/science.270.5242.1660 7502075

[B39] Rubio F. Schwarz M. Gassmann W. Schroeder J. I. (1999). Genetic selection of mutations in the high affinity K^+^ transporter HKT1 that define functions of a loop site for reduced Na^+^ permeability and increased Na^+^ tolerance. *J. Biol. Chem.* 274 6839–6847. 10.1074/jbc.274.11.6839 10066736

[B40] Rus A. Baxter I. Muthukumar B. Gustin J. Lahner B. Yakubova E. (2006). Natural variants of *AtHKT1* enhance Na^+^ accumulation in two wild populations of *Arabidopsis*. *PLoS Genet.* 2:e210. 10.1371/journal.pgen.0020210 17140289 PMC1665649

[B41] Schachtman D. P. Schroeder J. I. (1994). Structure and transport mechanism of a high-affinity potassium uptake transporter from higher plants. *Nature* 370 655–658. 10.1038/370655a0 8065452

[B42] Shahzad A. N. Pitann B. Ali H. Qayyum M. F. Fatima A. Bakhat H. F. (2015). Maize genotypes differing in salt resistance vary in jasmonic acid accumulation during the first phase of salt stress. *J. Agron. Crop Sci.* 201 443–451. 10.1111/jac.12134

[B43] Sunarpi H. T. Motoda J. Kubo M. Yang H. Yoda K. Horie R. (2005). Enhanced salt tolerance mediated by AtHKT1 transporter-induced Na^+^ unloading from xylem vessels to xylem parenchyma cells. *Plant J.* 44 928–938. 10.1111/j.1365-313X.2005.02595.x 16359386

[B44] Uozumi N. Kim E. J. Rubio F. Yamaguchi T. Muto S. Tsuboi A. (2000). The *Arabidopsis HKT1* gene homolog mediates inward Na^+^ currents in *Xenopus laevis* oocytes and Na^+^ uptake in *Saccharomyces cerevisiae*. *Plant Physiol.* 122 1249–1259. 10.1104/pp.122.4.1249 10759522 PMC58961

[B45] Very A. A. Sentenac H. (2003). Molecular mechanisms and regulation of K^+^ transport in higher plants. *Annu. Rev. Plant Biol.* 54 575–603. 10.1146/annurev.arplant.54.031902.134831 14503004

[B46] Wang T. T. Ren Z. J. Liu Z. Q. Feng X. Guo R. Q. Li B. G. (2014). SbHKT1;4, a member of the high-affinity potassium transporter gene family from Sorghum bicolor, functions to maintain optimal Na^+^ /K^+^ balance under Na^+^ stress. *J. Integr. Plant Biol.* 56 315–332. 10.1111/jipb.12144 24325391

[B47] Wang X. Wang H. Liu S. Ferjani A. Li J. Yan J. (2016). Genetic variation in ZmVPP1 contributes to drought tolerance in maize seedlings. *Nat. Genet.* 48 1233–1241. 10.1038/ng.3636 27526320

[B48] Wei Z. Y. Zhang Y. Y. Wang Y. P. Fan M. X. Zhong X. F. Xu N. (2016). Production of bioactive recombinant bovine chymosin in tobacco plants. *Int. J. Mol. Sci.* 17:624. 10.3390/ijms17050624 27136529 PMC4881450

[B49] Wisser R. J. Kolkman J. M. Patzoldt M. E. Holland J. B. Yu J. Krakowsky M. (2011). Multivariate analysis of maize disease resistances suggests a pleiotropic genetic basis and implicates a GST gene. *Proc. Natl. Acad. Sci. U.S.A.* 108 7339–7344. 10.1073/pnas.1011739108 21490302 PMC3088610

[B50] Xiang Y. Sun X. Gao S. Qin F. Dai M. (2016). Deletion of an endoplasmic reticulum stress response element in a ZmPP2C-A gene facilitates drought tolerance of maize seedlings. *Mol. Plant* 10 456–469. 10.1016/j.molp.2016.10.003 27746300

[B51] Xiang Y. Sun X. Gao S. Qin F. Dai M. (2017). Deletion of an endoplasmic reticulum stress response element in a ZmPP2C-A gene facilitates drought tolerance of maize seedlings. *Mol. Plant* 10 456–469. 10.1016/j.molp.2016.10.003 27746300

[B52] Yamaguchi T. Blumwald E. (2005). Developing salt-tolerant crop plants: challenges and opportunities. *Trends Plant Sci.* 10 615–620. 10.1016/j.tplants.2005.10.002 16280254

[B53] Yan H. Jia H. Chen X. Hao L. An H. Guo X. (2014). The cotton WRKY transcription factor GhWRKY17 functions in drought and salt stress in transgenic *Nicotiana benthamiana* through ABA signaling and the modulation of reactive oxygen species production. *Plant Cell Physiol.* 55 2060–2076. 10.1093/pcp/pcu133 25261532

[B54] Yan J. Kandianis C. B. Harjes C. E. Bai L. Kim E. H. Yang X. (2010). Rare genetic variation at *Zea mays* crtRB1 increases beta-carotene in maize grain. *Nat. Genet.* 42 322–327. 10.1038/ng.551 20305664

[B55] Yan J. Marilyn W. Jonathan C. (2011). Association mapping for enhancing maize (*Zea mays* L.) genetic improvement. *Crop Sci.* 51 433–449. 10.2135/cropsci2010.04.0233

[B56] Yang G. Yu L. Zhang K. Zhao Y. Guo Y. Gao C. (2017). A *ThDREB* gene from *Tamarix hispida* improved the salt and drought tolerance of transgenic tobacco and *T. hispida*. *Plant Physiol. Biochem.* 113 187–197. 10.1016/j.plaphy.2017.02.007 28222350

[B57] Zhu J. K. (2001). Plant salt tolerance. *Trends Plant Sci.* 6 66–71. 10.1016/S1360-1385(00)01838-0 11173290

[B58] Zorb C. Muhling K. H. Kutschera U. Geilfus C. M. (2015). Salinity stiffens the epidermal cell walls of salt-stressed maize leaves: is the epidermis growth-restricting? *PLoS One* 10:e0118406. 10.1371/journal.pone.0118406 25760715 PMC4356557

