# Supplementary figures and images for: Association Analysis and Identification of ZmHKT1;5 Variation With Salt-Stress Tolerance
Source: Front Plant Sci. 2018 Oct 12;9:1485. doi: 10.3389/fpls.2018.01485 (PMC6194160; doi:10.3389/fpls.2018.01485)

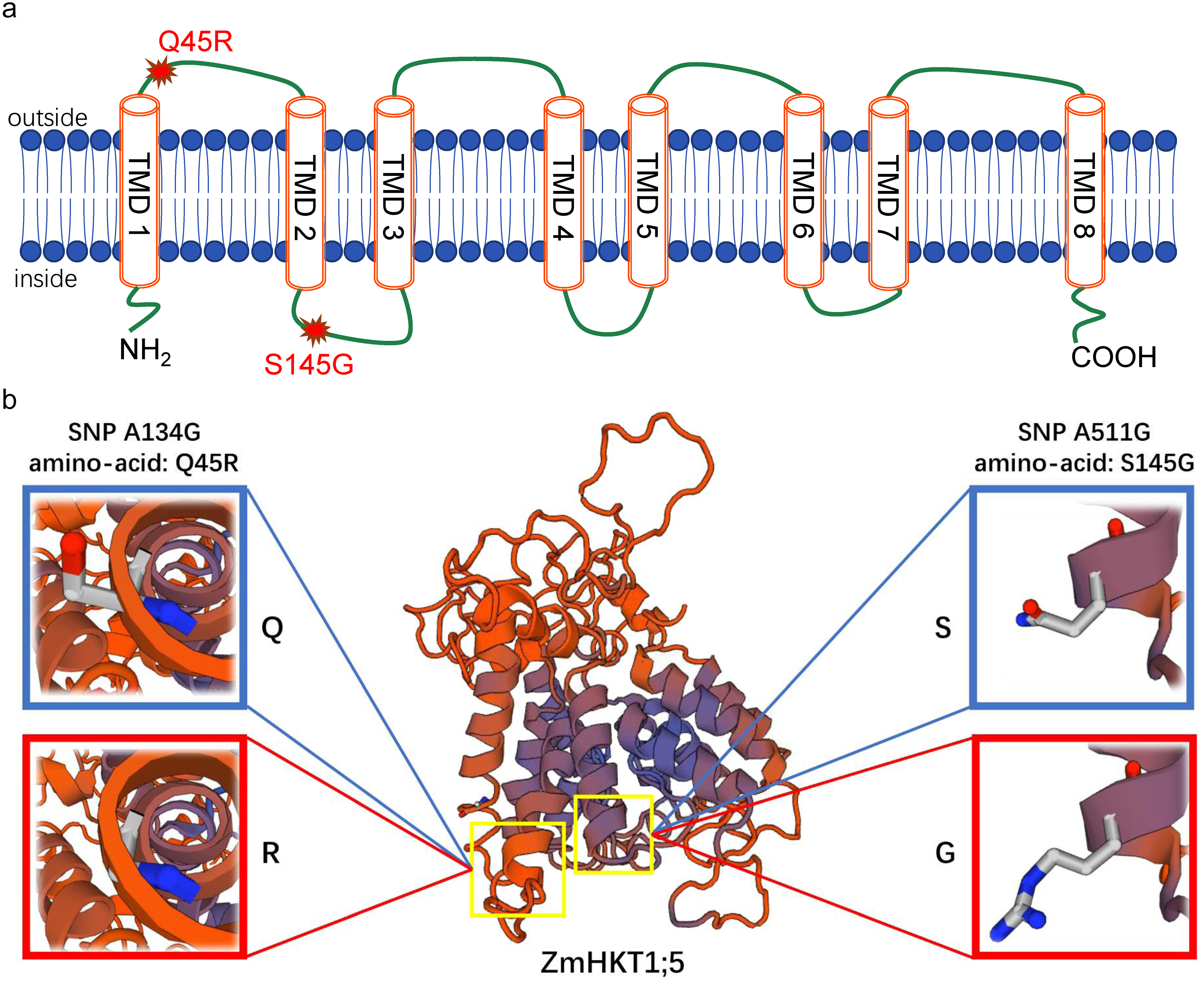

Supplement: FIGURE S1 — Structural model of ZmHKT1;5. (a) A model of the ZmHKT1;5 protein based on hydrophobicity plot analysis and the eight-TMD model reported previously (Kato et al., 2001). Asterisks indicate positions of residue substitution. (b) A protein structure diagram of ZmHKT1;5. SNP A134G and SNP A511G are marked. [file Image_1.TIFF]
