# Supplementary material for: Association Analysis and Identification of ZmHKT1;5 Variation With Salt-Stress Tolerance
Source: Front Plant Sci. 2018 Oct 12;9:1485. doi: 10.3389/fpls.2018.01485 (PMC6194160; doi:10.3389/fpls.2018.01485)
Supplement: TABLE S1 — Names and accession numbers of proteins used for phylogenetic analysis. [file Table_1.DOCX]

| **Table S1. Names and accession numbers of proteins used for phylogenetic analysis** | | | |
| --- | --- | --- | --- |
| Name | Species | Accession Number | Sequence Source |
| *AtHKT1;1* | *Arabidopsis thaliana* | AAF68393 | GenBank |
| *BdHKT* | *Brachypodium distachyon* | XP_003570995 | NCBI |
| *BoHKT1;1* | *Brassica oleracea* | AFI81996 | GenBank |
| *EcHKT1;1* | *Eucalyptus camaldulensis* | AAF97728 | GenBank |
| *EcHKT1;2* | *Eucalyptus camaldulensis* | AAD53890 | GenBank |
| *HvHKT1;5* | *Hordeum vulgare* | ABK58096 | GenBank |
| *HvHKT2;1* | *Hordeum vulgare* | CAJ01327 | GenBank |
| *McHKT1;1* | *Mesembryanthemum crystallinum* | AAK52962 | GenBank |
| *McHKT1;2* | *Mesembryanthemum crystallinum* | AAO73474 | GenBank |
| *MtHKT1:1* | *Medicago truncatula* | XP_003620952 | NCBI |
| *OsHKT1;1* | *Oryza sativa* | CAD37183 | GenBank |
| *OsHKT1;3* | *Oryza sativa* | CAD37185 | GenBank |
| *OsHKT1;4* | *Oryza sativa* | CAD37197 | GenBank |
| *OsHKT1;5* | *Oryza sativa* | BAB93392 | GenBank |
| *OsHKT2;1* | *Oryza sativa* | BAB61789 | GenBank |
| *OsHKT2;2* | *Oryza sativa* | BAB61791 | GenBank |
| *OsHKT2;3* | *Oryza sativa* | CAD37187 | GenBank |
| *OsHKT2;4* | *Oryza sativa* | CAD37199 | GenBank |
| *PhaHKT2;1* | *Phragmites australis* | BAE44385 | GenBank |
| *PtHKT1;1* | *Populus trichocarpa* | XP_002325229 | GenBank |
| *PutHKT2;1* | *Puccinellia tenuiflora* | ACT21087 | GenBank |
| *SabHKT1;1* | *Salicornia bigelovii* | ADG45565 | GenBank |
| *SbHKT1:3* | *Sorghum bicolour* | Sb04g005010 | Phytozome |
| *SbHKT1:4* | *Sorghum bicolour* | Sb06g027900 | Phytozome |
| *SbHKT1:5* | *Sorghum bicolour* | Sb03g012590 | Phytozome |
| *SbHKT2;1* | *Sorghum bicolour* | Sb10g029000 | Phytozome |
| *SsHKT1;1* | *Suaeda salsa* | AAS20529 | GenBank |
| *TaHKT1;5-B1* | *Triticum aestivum* | ABG33947 | GenBank |
| *TaHKT1;5-B2* | *Triticum aestivum* | ABG33948 | GenBank |
| *TaHKT1;5-D* | *Triticum aestivum* | ABG33945 | GenBank |
| *TaHKT2;1* | *Triticum aestivum* | AAA52749 | GenBank |
| *TmHKT1;4-A1* | *Triticum monococcum* | ABK41858 | GenBank |
| *TmHKT1;4-A2* | *Triticum monococcum* | ABK41857 | GenBank |
| *TmHKT1;5-A* | *Triticum monococcum* | ABG33946 | GenBank |
| *TsHKT1;1* | *Thellungiella salsuginea* | AFJ23835 | GenBank |
| *TsHKT1;2* | *Thellungiella salsuginea* | ABK30935 | GenBank |
| *ZmHKT1;5* | *Zea mays* | GRMZM2G047616 | Phytozome |
| *ZmHKT2;1* | *Zea mays* | GRMZM2G135674 | Phytozome |
| *VvHKT1:1* | *Vitis vinifera* | CBI40132 | GenBank |
| *VvHKT1:2* | *Vitis vinifera* | CBI40134 | GenBank |
