# Supplementary material for: Association Analysis and Identification of ZmHKT1;5 Variation With Salt-Stress Tolerance
Source: Front Plant Sci. 2018 Oct 12;9:1485. doi: 10.3389/fpls.2018.01485 (PMC6194160; doi:10.3389/fpls.2018.01485)
Supplement: TABLE S2 — Tolerance to salt stress of different inbred lines. [file Table_2.DOCX]

| **Table S2 Tolerance to salt stress of different inbred lines** | | | |
| --- | --- | --- | --- |
| Inbred line | Salt tolerance level | Inbred line | Salt tolerance level |
| 434 | MR | J853 | R |
| 444 | MR | K10 | S |
| 835 | R | K12 | MR |
| 8902 | S | K22 | S |
| 78599 | S | L105 | S |
| 81162 | R | LX9801 | MR |
| A188 | HS | Mo17 | S |
| B0049 | S | N178 | S |
| B73 | S | N5 | R |
| BM | MR | P138 | S |
| C72 | S | Pa91 | R |
| C7-2 | R | Q318 | MR |
| C8605-2 | HR | S0073 | MR |
| D1324 | MR | S137 | S |
| D340 | HS | S139 | HS |
| D360 | S | S144 | S |
| D598 | HS | S273 | S |
| D9046 | S | S287 | HR |
| H344 | MR | T7922 | R |
| H8723 | R | U8112 | MR |
| H99 | HS | Ye478 | HS |
| HC | MR | Z106 | MR |
| HZ-4 | MR | Z22 | R |
| J1037 | MR | Z31 | MR |
| J163 | MR | Z330 | S |
| J5918 | MR | Z451 | R |
| J818 | MR | Zheng58 | HR |
| HR: Highly salt-resistant, R: Salt-resistant, MR: Moderately salt-resistant, S: Salt-sensitive, HS: Highly salt-sensitive. | | | |
